# Clinical Onset and Multiple Sclerosis Relapse after SARS-CoV-2 Infection

**DOI:** 10.3390/neurolint13040066

**Published:** 2021-12-06

**Authors:** Antonia Pignolo, Maria Aprile, Cesare Gagliardo, Giovanni Maurizio Giammanco, Marco D’Amelio, Paolo Aridon, Giuseppe La Tona, Giuseppe Salemi, Paolo Ragonese

**Affiliations:** 1 Department of Biomedicine, Neuroscience and Advanced Diagnostics (BiND), University of Palermo, Via Gaetano la Loggia n.1, 90129 Palermo, Italy; niettapignolo@gmail.com (A.P.); dr.aprilemaria@gmail.com (M.A.); cesare.gagliardo@unipa.it (C.G.); marco.damelio@unipa.it (M.D.); paolo.aridon@unipa.it (P.A.); giuseppe.latona@unipa.it (G.L.T.); 2 Department of Health Promotion, Mother and Child Care, Internal Medicine and Medical Specialties (ProMISE), University of Palermo, 90129 Palermo, Italy; giovanni.giammanco@unipa.it

**Keywords:** multiple sclerosis, COVID-19, relapse, onset, vaccine

## Abstract

Severe acute respiratory syndrome coronavirus 2 (SARS-CoV-2) infection has been associated with several neurological disorders including headache, facial palsy, encephalitis, stroke, demyelinating disorders. The present report will discuss cases of multiple sclerosis (MS) onset and relapse both beginning early after SARS-CoV-2 infection. In both cases, magnetic resonance imaging (MRI) showed widespread bilateral subcortical and periventricular active lesions. Serum IgG against SARS-CoV-2 Spike antigens confirmed seroconversion with titers that are considered not definitely protective against possible reinfection. We hypothesize that SARS-CoV-2 infection, as previously reported for other viruses, could drive an active inflammatory response that can contribute either to the onset of MS or its relapse. The presented data further support the importance of vaccination in individuals with MS.

## 1. Introduction

Multiple sclerosis (MS) is an immune-mediated disorder of the central nervous system (CNS) and one of the most common causes of disability in young adult patients. MS is characterized by demyelination and axonal degeneration leading to progressive CNS damage and accumulation of persistent disability [1]. The etiology of MS has been widely investigated through immunobiology, genetics, and epidemiology. The autoimmune hypothesis involves dis-regulation of auto-reactive T cells with B lymphocytes and macrophages, in which virus-induced immunopathology plausibly play a relevant role [1,2]. MS exacerbation, characterized by the worsening of known symptoms or by emerging new symptoms lasting over 24 h after a period of disease stability with increasing scoring in the Expanded Disability Status Scale (EDSS), could be caused by systemic infection, especially upper respiratory tract infection or flu. Indeed, the risk of relapse increased during a predefined at-risk period (ARP) between 2 weeks before and up to nearly 2 months after the infection in previous reports [3]. Evidence of inflammatory activation was supported by higher plasma concentrations of soluble intracellular adhesion molecules [1] and specific chemokines (XCL1, CXCL5, CXCL8, CCL27, CCL28, CXCL12, CX3CL1, and IFN-γ), which increased in cerebrospinal fluid (CSF) in MS patients and up-regulated in CNS during infections and exacerbation [4].

In December 2019, a new pathogen, belonging to the coronaviruses family, was identified in Wuhan (China) as the cause of an illness later designated by the World Health Organization as COVID-19 [5]. The severe acute respiratory syndrome coronavirus 2 (SARS-CoV-2) is responsible for a wide spectrum of infection severity, whose main manifestation is pneumonia. Neurologic complications have been reported in patients with COVID-19. Myalgia, headache, dizziness, anosmia and dysgeusia were identified in early stages and in patients with mild disease. Less frequently myopathy, dysautonomia, cerebrovascular disease, movement disorders, seizures, encephalitis, Guillain-Barré syndrome, acute demyelinating encephalomyelitis (ADEM) and optic neuritis have been reported [6] Several reports provided evidence for the neurotropism of SARS-CoV-2 [7]. Recent evidences suggest an increasing risk for demyelinating lesions of the CNS [8]. To date, the correlation between viral infections and MS is still not entirely clear and a possible role as trigger or co-factors in the onset and development of the disease has been hypothesized [9]. Although the oldest historical hypotheses suggested that exposure to some vaccines might represent an immune stimulus triggering the onset of MS, this was not confirmed by a considerable number of studies [10]. Conversely, the link between influenza infection and increased relapse rate, has been described as well as increased mortality [4], whereby influenza vaccine is recommended annually in these patients [11]. Similarly, evidence shows that COVID-19 induces immune-mediated processes leading to an increased risk of neurological damage [12]. In the present report, we describe two cases of patients, showing MS disease onset and MS relapse after COVID-19 infection, respectively. We will also discuss the relationship between viral infections and acute MS relapse in the perspective of preventive strategies, like mass immunization, in order to prevent disease exacerbation and disability accrual as consequences of infections.

## 2. MS Onset

**Figure 1:**
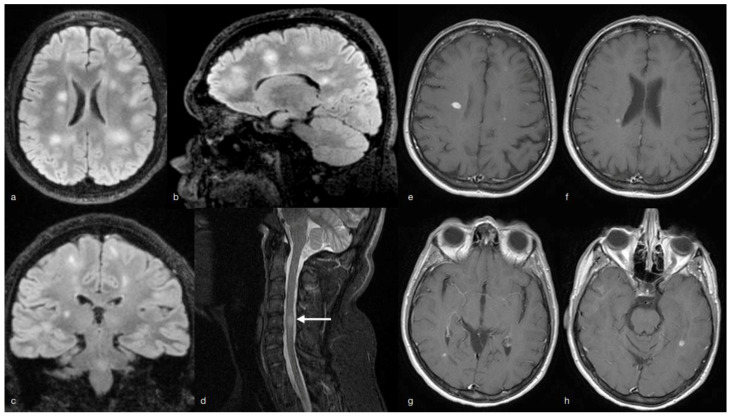
Multiple subcortical, perivenular and periventricular hyperintense lesions in FLAIR (**a**–**c**). A paramedian intramedullary with swollen C5 hypertense lesion in STIR (**d**). Case 2: active lesion with enhancement in T1 gad + (**e**–**h**).

Case 1. The first patient was a 21-year-old man with a medical history of cardiac surgery for an atrioventricular septal defect, with no other significant anamnestic data or medications taken by the patient. In late October 2020 the father, who cohabitated with the patient, developed COVID-19. After 1 week the patient started complaining of fever, headache, anosmia and dysgeusia, followed few days later, by hand paresthesia and fatigue, attributed by the general practitioner to COVID-19. At the end of December 2020, he developed a right facial nerve palsy. The neurological examination showed brisk tendon reflexes at four limbs and a central involvement of the seventh right cranial nerve. Cerebral and spine MRI revealed bilateral non-enhancing white matter perivenular lesions with blurred edges involving the periventricular and subcortical areas as well as the corpus callosum and the brain stem with a swollen spine lesion in C5. The patient was treated with intravenous steroids for five consecutive days with partial recovery of the sensorial symptoms and the facial palsy. At the end of January, he was admitted to our hospital where a new MRI examination confirmed the previous findings with no active brain or spine lesions (Figure 1a–d). A diagnostic lumbar puncture showed elevated CSF proteins (574 mg/L with cut-off 150–450), increased Link index, 78/mmc cells (above 95% lymphocytes) and more than six unmatched oligoclonal IgG bands. The SARS-CoV-2 PCR in the CSF was negative (SARS-CoV-2 ELITe MGB^®®^ CE-IVD Kit targeting ORF8-gene and RdRp-gene), while he tested positive for anti-SARS-CoV-2 Spike Ags IgG, (52.7 AU/mL IgG; cut-off: 15 AU/mL) with negative IgM, supporting the recent SARS-CoV-2 infection. Other serological and CSF tests for viruses, including also Epstein–Barr virus (EBV), herpes simplex virus (HSV), and cytomegalovirus (CMV), were negative. Autoimmunity testing did not show any pathological result. Anti-MOG and anti-AQP4 were also negative. Brainstem auditory (BAEP) evoked responses, visual evoked responses (VEPs), somatosensory evoked potentials (SSEP) and motor evoked potentials (MEPs) proved normal. At the follow-up visit neurological evaluation showed only brisk tendon reflexes and the barely visible sequelae of the facial nerve palsy (EDSS score 1.5). At the end of the diagnostic work-up, the patient, considering also the cardiological comorbidity, was started on pegylated beta interferon. 

## 3. Multiple Sclerosis (MS) Relapse

Case 2. The second patient is a 52-year-old woman affected by MS since the age of 22, who started cladribine therapy at the end of July 2020. Her EDSS score at that time was 1.5. On August, she had the second month cladribine administration and as planned, in October she underwent blood chemistry tests showing a decreased total lymphocyte count (780 × 10^3^/μL) with CD19 3%, CD4 52% and CD8 29%. On 28 October 2020, she developed symptoms consistent with COVID-19 (mild acute respiratory symptoms, chills, joint and muscle pain, hyposmia, ageusia and fever not exceeding 37.5 °C) lasting for about 2 weeks and followed by persisting fatigue. A serological test for COVID-19 disease, performed in January 2021, showed positive IgG titers of 40.2 AU/mL (cut-off: 15 AU/mL) and negative IgM (0.17 AU/mL). Although the SARS-CoV-2 infection determined mild symptoms, the patient reported increased fatigue beginning in the weeks following the infection, determining slightly reduced daily living performance. In January 2021 (2 months after COVID-19) the follow-up MRI, showed multiple new active lesions in the supratentorial white matter (Figure 1e–h); few days later her motor symptoms further worsened and she complained of mild clumsiness on her right side together with slight strength impairment. Symptoms fully recovered after methylprednisolone at the dose of 1000 mg for 5 consecutive days.

## 4. Discussion

The cases described are examples of post-infectious MS onset or relapse. These cases are in line with previous observations reporting the association between viral infections and demyelinating diseases onset or relapse [9]. Worldwide, several studies have been conducted to investigate the pathological bases of neurological disorders associated to viral infections. Despite it not being fully clarified if viral infections may directly or indirectly concur with the development of neurological diseases, evidence showed a strong association between infections and disorders like MS, epilepsy, Parkinson’s disease, Alzheimer’s disease and Guillain-Barré syndrome. Pathogens like influenza viruses, EBV, varicella-zoster virus (VZV), CMV, human herpesvirus 6 (HHV-6), and human herpesvirus 7 (HHV-7) have been associated, in particular, with MS. The MS etiology is multifactorial and viral infections seem to be a significant environmental risk factor [13]. Decades of investigations have linked viruses to both onset and progression of MS, proposing the concept of viral infections as a trigger of MS relapse or alternatively, viral activation as an epiphenomenon during relapses. Interestingly, several pathogens have been associated with disease activity, while vaccinations have not [14]. A recent report described a case series of MS symptoms worsening occurring after vaccination against COVID-19. However, the authors underlined that in their case series there was no background knowledge of the amount of patients who underwent vaccination in the same period. Moreover, they did not perform comparisons with the rate of relapse among patients who did not receive COVID-19 vaccination [15]. In our MS center, with over more than 300 individuals with MS who underwent COVID-19 vaccination, we observed only one possible disease relapse occurring during the month following vaccination. Recently, a study evaluated the relapse rate in the MS population who underwent COVID-19 vaccination, compared to a cohort of non-vaccinated patients, showing no increased risk of relapses [16]. Similarly, an Italian prospective, self- controlled, multicentric observational study conducted in 25 Italian MS centers, concluded that Pfizer/BioNTech BNT162b2 vaccine did not increase the short-term risk of clinical reactivation in MS patients [17].

A literature review for demyelinating disorders showed very few reports, mostly observing new MRI demyelinating lesions, ADEM and an optical neuritis after SARS-CoV-2 infection [8,18,19]. Another study reported MS worsening occurring in three patients after COVID-19 infection without a clear confirmation of a preceding relapse. The authors concluded that MS worsening could be attributable to pseudo-relapses associated to acute infection [20]. In the present report, we discuss two cases, one with the onset of MS, and the second who relapsed after SARS-CoV-2 infection. The first case had the onset of a demyelinating disorder whose temporal and spatial dissemination criteria fit with the McDonald revised MS criteria. The CSF pleocytosis could raise doubts about a relapsing ADEM, although the clinical onset and the MRI pattern are not supportive of such a diagnosis. The cases we described here deserve some considerations. We point out that the clinical onset occurred within a few weeks from the preceding SARS-CoV-2 disease confirming that, according to previous literature, COVID-19 infection might also represent a trigger for onset or relapse of MS. The second case we reported showed how, even in the presence of a lymphocyte NADIR occurring after cladribine therapy, clinical symptoms of SARS-CoV-2 infection could be very mild and followed by the development of neutralizing antibodies against SARS-CoV-2. Despite a lymphocytes decrease apparently indicating a response to cladribine therapy, in this patient the COVID-19 disease was followed by MS re-activation. Finally, as previously reported [21], serum IgG titers against SARS-CoV-2 that we observed did not reach clearly protective values. This is of particular interest and strengthens the concept that vaccination should be recommended also in individuals who already had SARS-CoV-2 infection since the observed Ig values do not necessarily imply disease immunity and, as already reported, these values tend to decrease with time. Viral infections have long been considered as possible factors associated to MS pathological mechanisms or as triggers for MS relapse [9]. Recent clinical studies of COVID-19 disease have investigated the role of extracellular vesicles (EVs), already known to be involved in cell-to-cell communication, also between components of the blood–brain barrier (BBB). Microvesicles (MVs) and exosomes are involved both in viral infection and antiviral response. Recent evidence suggests a role of exosomes in SARS-CoV-2 infection due to their ability to transfer the ACE2 receptor to cells exposed to viral docking, supporting virus internalization and infection spread [22]. Furthermore, evidence regarding neuroinflammation highlighted that MVs would promote BBB injury through co-involvement of tumor necrosis factor-α (TNF-α) and interleukin-6 (IL-6) which have been reported as increased during COVID-19 infection [23].

## 5. Conclusions

In the present emergency due to the SARS-CoV-2 pandemic, there still appear to be some vaccine hesitancies the reasons for which derive from the fear both of possible side effects of vaccines and of possible effects that a vaccine might have on MS disease course. Actually, available data for Pfizer/BioN-Tech BNT162b2 vaccine in the MS population and results did not support evidence for an association between vaccination and risk for MS onset or relapses [16,17]. Conversely, the present report confirms the risk, after SARS-Cov-2 infection, of demyelinating events including MS onset and relapse. Hence, we conclude that given the unpredictable disease course in patients who develop SARS-CoV-2 infection and the risk for relapses determined by COVID-19 disease compared to the low rate of relapses after vaccination, immunization against SARS-COV-2 should be recommended for individuals with MS.

## Data Availability

All data are included in this article. Further enquiries can be directed to the corresponding author.
